# Digital Twin and Artificial Intelligence Technologies to Assess the Type IA Endoleak

**DOI:** 10.3390/bioengineering13010001

**Published:** 2025-12-19

**Authors:** Sungsin Cho, Hyangkyoung Kim, Jinhyun Joh

**Affiliations:** 1 Department of Surgery, Kyung Hee University Hospital at Gangdong, Kyung Hee University School of Medicine, Seoul 05278, Republic of Korea; 01ssunny@naver.com; 2 Department of Surgery, Ewha Womans University College of Medicine, Ewha Womans University Medical Center, Seoul 07985, Republic of Korea; cindycrow7456@gmail.com

**Keywords:** aortic aneurysm, abdominal, endovascular aneurysm repair, endoleaks, digital twin, artificial intelligence

## Abstract

Background/Objectives: Endovascular aneurysm repair (EVAR) is the standard treatment for abdominal aortic aneurysms, but the risk of endoleak compromises its effectiveness. Type IA endoleak, stemming from an inadequate proximal seal, is the most critical complication associated with the highest risk of rupture. Current preoperative planning relies on static anatomical measurements from computed tomography angiography that fail to predict seal failure due to dynamic biomechanical forces. This study aimed to retrospectively validate the predictive accuracy of a novel physics-informed digital twin and artificial intelligence (AI) model for predicting type IA endoleak risk compared to conventional static planning methods. Methods: This was a retrospective, single-center proof-of-concept validation study involving 15 patients who underwent elective EVAR (5 with confirmed type IA endoleak and 10 without type IA endoleak). A patient-specific digital twin was created for each case to simulate stent-graft deployment and capture the dynamic biomechanical interaction with the aortic wall. A logistic regression AI model processed over 16,000 biomechanical measurements to generate a single, objective metric of the endoleak risk index (ERI). The predictive performance of the ERI (using a cutoff of 0.80) was assessed and compared against a 1:3 propensity score-matched conventional control group (*n* = 45) who received traditional anatomical-based planning. Results: The mean ERI was significantly higher in the endoleak-positive group (0.85 ± 0.10) compared to the endoleak-negative group (0.39 ± 0.11) (*p* = 0.011). The digital twin/AI model demonstrated superior predictive capability, achieving an overall accuracy of 80% (95% CI: 51.9–95.7) and an area under the curve (AUC) of 0.85 (95% CI: 0.58–0.99). Crucially, the model achieved a sensitivity of 100% and a negative predictive value (NPV) of 100%, correctly identifying all high-risk cases and ruling out endoleak in all low-risk cases. In stark contrast, the matched conventional planning group achieved an overall accuracy of only 51.1% and an AUC of 0.54. Conclusion: This physics-informed digital twin and AI framework successfully validated its capability to accurately and objectively predict the risk of type IA endoleak following EVAR. The derived ERI offers a significant quantitative advantage over traditional static anatomical measurements, establishing it as a highly reliable safety tool (100% NPV) for ruling out endoleak risk. This technology represents a critical advancement toward personalized EVAR planning, enabling surgeons to proactively identify high-risk anatomies and adjust treatment strategies to minimize post-procedural complications. Further large-scale, multicenter prospective trials are necessary to confirm these findings and support clinical adoption.

## 1. Introduction

Endovascular aneurysm repair (EVAR) has fundamentally reshaped the landscape of care for patients with abdominal aortic aneurysms (AAA), offering a minimally invasive and highly effective alternative to traditional open surgical repair [1,2,3]. By excluding the aneurysm sac from systemic blood flow, EVAR mitigates the risk of aneurysm rupture and has demonstrated favorable outcomes. However, the procedure is not without its specific challenges, primarily the risk of endoleak [4]. Endoleaks, defined as blood flow into the aneurysm sac outside the lumen of the stent-graft, are a major source of post-procedural morbidity and mortality [5]. They can compromise the integrity of the repair, leading to sustained aneurysm sac pressurization and, in some cases, re-expansion and rupture [6]. Among the various types of endoleaks, the type IA is considered the most critical, as it stems from an inadequate seal at the proximal landing zone of the stent-graft and is associated with the highest risk of rupture [7]. Type IA endoleaks are a serious complication, with an incidence rate affecting up to 30% of patients and a rupture risk as high as 7.5% within two years [8]. The clinical significance of this complication underscores the urgent need for robust preoperative strategies to identify and mitigate this risk.

Current planning for EVAR relies heavily on preoperative computed tomography angiography (CTA) and standardized measurements. The limitations of conventional planning tools represent the most significant barrier to optimizing outcomes in EVAR. Current preoperative assessments are overwhelmingly dependent on static, two-dimensional measurements derived from CTA. While these tools are essential for basic sizing, they fundamentally fail to capture the dynamic, patient-specific biomechanical realities of stent-graft deployment [9]. The complex, non-linear deformation of the aorta, combined with the radial forces exerted by the stent-graft, can lead to subtle but significant gaps at the landing zones that are not apparent on static CTA images [10]. Furthermore, even these static measurements suffer from considerable interobserver and intraobserver variability, potentially leading to differing endograft sizing and selection among surgeons [11,12]. Consequently, reliance on static geometry leads to a critical gap in prediction. These conventional methods cannot accurately foresee where and when a seal failure will occur. This inability is directly responsible for the persistence of type IA endoleaks, as current planning often yields a dimensionally successful procedure that still results in a seal failure because the dynamic forces were misjudged. This forces clinicians to rely on general guidelines and subjective experience rather than precise, patient-specific data. The core problem, therefore, is the lack of a quantitative, predictive tool that can simulate the post-deployment seal failure preoperatively, leading to suboptimal treatment planning, the need for extensive long-term surveillance, and preventable secondary interventions.

To overcome the inherent limitations of static anatomical assessments, the application of personalized computational modeling is rapidly emerging. A digital twin and artificial intelligence (AI) model are a patient-specific, three-dimensional computational model of the aortic aneurysm that integrates the unique biomechanical properties of the patient’s aortic tissue and the design characteristics of a commercially available stent-graft. This sophisticated model allows for a precise simulation of stent-graft deployment and its sealing effectiveness, offering a level of predictive insight that is unattainable with current methods [13]. Deriche L et al. demonstrated the usefulness of patient-specific numerical simulation of EVAR to predict type IA endoleak [14]. However, this system requires specialized expertise and extreme computational intensity from the numerous patients’ dataset. A proof-of-concept (POC) is a small, focused project designed to verify the technical feasibility and potential value of an AI for a specific problem.

The lack of a reliable, objective, and predictive tool that can simulate this intricate mechanical interaction represents a substantial clinical challenge that impacts patient safety and procedural efficiency. The purpose of this study is to retrospectively validate the predictive accuracy of a novel digital twin and AI model by comparing its simulated outcomes against the actual postoperative results of patients who have undergone EVAR and to assess the model’s performance metrics, establish its quantifiable advantage over conventional planning.

## 2. Methods

### 2.1. Study Design and Patient Cohort

This retrospective, single-center, POC validation study included 15 patients who underwent elective EVAR for infrarenal AAA. Patient selection was based on the availability of preoperative CTA scans with sufficient resolution for three-dimensional (3D) reconstruction. All patients had a confirmed infrarenal AAA and were deemed suitable candidates for EVAR by the treating physicians. The cohort included 10 patients who were confirmed to be free of a type IA endoleak after the procedure, and 5 patients who were clinically diagnosed with a type IA endoleak, as confirmed by follow-up CTA imaging. Data included demographic information, aneurysm characteristics (diameter, length, tortuosity), and the type and dimensions of the deployed stent-graft. The study protocol was reviewed by the Institutional Review Board (IRB) of Kyung Hee University Hospital, and the need for individual patient consent was waived due to the retrospective, de-identified nature of the data.

### 2.2. Algorithm Type, Feature Selection Process, and Training

The AI component utilized in this study is a logistic regression model as a supervised machine learning (ML) algorithm. We selected this algorithm due to its interpretability, robustness with a limited number of derived features, and its effectiveness in providing a probability of a binary outcome (endoleak positive/negative). The features provided to the AI are derived exclusively from the quantitative measurements generated by the virtual stent-graft deployment simulation. The present study is a retrospective feasibility study using a small, single-center cohort. Therefore, this cohort was used solely for model validation and testing against known clinical outcomes, not for initial training. The initial training and hyperparameter tuning of the logistic regression model were performed on a separate, anonymized, simulated dataset.

### 2.3. Digital Twin Model Creation

**Figure 1:**
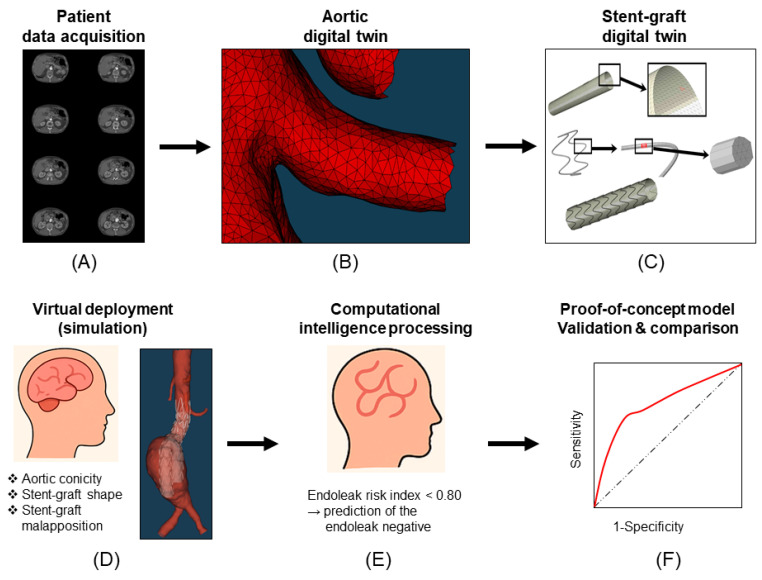
Pipeline of the digital twin and artificial intelligence model for endoleak prediction. Patient data acquisition: (**A**) The process begins with preoperative computed tomography angiography (CTA) images, which provide the static anatomical input required for modeling. (**B**) Aortic digital twin: A high-fidelity, patient-specific 3D geometric model of the aorta is generated from the CTA data. The geometry is transformed into a finite element mesh for subsequent biomechanical analysis, incorporating material properties of the aortic wall. (**C**) Stent-graft digital twin: The specific endograft planned for the patient is digitally characterized, replicating its geometry and material properties to simulate its superelastic behavior upon deployment. (**D**) Virtual Deployment (Simulation): The two digital twins are combined, and a simulation is performed to model the complex, dynamic expansion and interaction of the stent-graft within the patient’s aorta. The simulation focuses on quantifying key biomechanical features in the sealing zone, including aortic conicity, stent-graft shape, and stent-graft malapposition. (**E**) Computational intelligence processing: An automated algorithm processes the measurements generated by the simulation to calculate the endoleak risk index (ERI). A score, such as ERI < 0.80, is used to generate a binary prediction, such as the prediction of endoleak negative. (**F**) Proof-of-concept model validation & comparison: The model’s predictive performance is validated retrospectively against actual clinical outcomes using metrics like the area under the curve. This step assesses the model’s ability to accurately discriminate between endoleak-positive and endoleak-negative cases.

A patient-specific digital twin model was created for each patient using their preoperative CTA data (Figure 1A). The process began with the segmentation of the aortic anatomy, from the suprarenal aorta down to the iliac arteries, to generate a precise 3D geometric model. This segmentation was performed using specialized software The Vascular Modeling Toolkit (VMTK 1.4.0) to meticulously delineate the aortic wall and the aneurysm sac (Figure 1B). Once the geometry was established, a finite element mesh was generated, discretizing the continuous aortic wall into a network of small, interconnected elements. This meshing process is critical for enabling subsequent biomechanical simulations. The model’s fidelity was further enhanced by incorporating the specific material properties of the patient’s aortic wall. The aorta’s tissue is known to be non-linear, anisotropic, and viscoelastic. To account for its anisotropic nature, a specific constitutive model was used, which defines the material’s elastic properties in different directions. The formula used to model the orthotropic properties of the aortic wall is given by:

1/E_θ_ = cos^4^θ/E_L_ + sin^4^θ/E_C_ + 1/4(1/G_LC_ − 2v_LC_/E_L_)sin^2^(2θ)

where E_θ_ represents the elastic modulus at an angle θ relative to the longitudinal direction, E_L_ is the longitudinal elastic modulus, E_C_ is the circumferential elastic modulus, G_LC_ is the shear modulus, and v_LC_ is Poisson’s ratio. This formula ensures that the model accurately reflects the differential stiffness of the aortic tissue, which is stiffer in the longitudinal direction than the circumferential direction due to the orientation of collagen and elastin fibers.

### 2.4. Stent-Graft Characterization

For each patient, the specific stent-graft used in their EVAR procedure was digitally characterized and incorporated into their respective digital twin model. This involved creating a 3D model of the stent-graft that accurately replicated its geometry, including the wireframe structure, fabric material, and dimensions (Figure 1C). The material properties of the stent-graft’s components were defined based on manufacturer data. The constitutive models for these materials were calibrated to represent their superelastic or pseudoelastic behavior, which is crucial for accurately simulating the radial force exerted on the aortic wall upon deployment. This step ensured that the virtual interaction between the stent-graft and the patient’s anatomy was as realistic as possible, allowing for a precise prediction of the final deployed state.

### 2.5. Virtual Deployment and Analysis

The core of this system is Finite Element Analysis (FEA), which performs a rigorous, physics-based mechanical simulation of the device deployment. This FEA process model large deformation and complex contact mechanics, capturing the dynamic expansion and precisely quantifying the final sealed configuration. Using a high-fidelity FEA solver, the digital stent-graft was virtually released from its delivery catheter within the model. This process captured the complex, non-linear deformation of both the stent-graft and the aneurysm sac as the device expanded to its final configuration (Figure 1D). The primary focus of this simulation was to analyze the sealing integrity at the proximal landing zone, specifically identifying any potential gaps between the stent-graft and the aortic wall that would indicate a high probability of a type IA endoleak. The simulation also provided data on the stress and strain distribution on the aortic wall, offering additional biomechanical insights into the postoperative state. An AI component was critically integrated into this phase to transform the massive, high-resolution biomechanical data generated by the FEA simulation into actionable clinical metrics. This AI algorithm was essential for automated feature extraction and quantification, processing raw simulation data across numerous measurements (such as 16,000 radius measures performed per patient). The AI’s role was to quantify specific geometric and mechanical features, including local apposition, oversizing, and the precise location and extent of stent-graft malapposition (SGM), which represents the precise gap between the device and the aortic wall. This automated analysis ensures objective and consistent data processing for all patients, which is unattainable with manual or static imaging methods. The culmination of the analysis was the generation of a single, objective risk score. This score was derived from the specific geometric and apposition features quantified by the AI component. The model’s final output was a binary prediction of “endoleak positive” or “endoleak negative”. Finally, the virtual deployment simulations and the resulting predicted final configuration of the graft were visually compared with intraoperative fluoroscopic images and postoperative CTA images to confirm the model’s ability to accurately predict the ultimate clinical outcome.

### 2.6. Computational Intelligence for Feature Extraction

**Figure 2:**
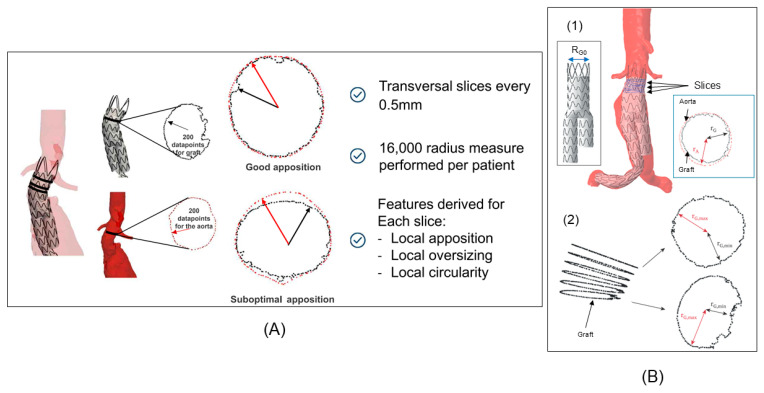
Local aortic-graft apposition analysis and calculation of endoleak risk index. (**A**) It displays a cross-sectional view of the aorta-graft interface, highlighting the concept of local apposition, oversizing, and circularity. Aorta and graft transversal slices were performed every 0.5 mm along the first two covered stents. Aorta and graft circumferences were discretized into dots (with around 200 dots per circumference). A total of 16,000 radius measurements were performed per patient. (**B**) Digital twin proximal sealing area post-analysis. (1) R_G0_ was defined as initial proximal stent graft diameter. Distance between the center of aorta and graft along the centerline and circumference dot were performed for the aorta (rA) and graft (rG) along the entire circumference. (2) Minimum (rG,min) and maximum (rG,max) graft radii were extracted for each slice.

The massive, high-resolution output dataset generated by the FEA simulation was processed by a customized computational intelligence (CI) algorithm, functioning as an automated feature extraction tool. This step directly addresses the challenge of interpreting complex simulation data and forms the AI component of the study. The CI algorithm automatically performs over 16,000 radial measurements across multiple cross-sectional slices within the proximal aortic neck (Figure 2). This algorithm’s primary role is sophisticated feature engineering, systematically quantifying the geometric and biomechanical interaction to derive three physics-informed features including aortic conicity (AC) that is quantified the degree of taper or widening of the aortic neck, calculated from the measured radii at the supra-renal and infra-renal boundaries and SGM and stent-graft shape (SGS) which is measured the deviation of the deployed stent-graft’s cross-section from a perfect circle, indicative of internal structural stress or external confinement forces.

### 2.7. Calculation of the Endoleak Risk Index

The Endoleak Risk Index (ERI) was conceived as a single, quantitative metric to integrate the multiple biomechanical risk factors identified by the simulation and AI component [14]. It represents the probability of a type IA endoleak occurring based on the patient’s unique anatomy and the virtualized stent-graft performance. To establish this index, a set of key features that exhibited a high correlation with the clinical outcome of endoleak was selected, focusing primarily on geometric anomalies and quantifiable measures of stent-graft apposition derived from the FEA simulation data. The ERI is a composite score calculated by aggregating and weighting these critical risk factors as follows: ERI = AC + SGS_2_ + (SGM_2_)/2, where AC represents aortic conicity, which quantifies the geometric taper of the sealing zone, SGS_2_ represents sum of differences in all slices related to the stent-graft shape, which assesses how the device deforms into an optimal sealing configuration, and SGM_2_) represents maximum length of stent-graft malapposition, which precisely measures the size and location of any gaps between the aortic wall and the device. Each factor was included with a specific coefficient, determined through preliminary analysis and optimization, to reflect its relative contribution to the final endoleak risk. The final ERI is a normalized, dimensionless value, typically ranging from 0 to 1, facilitating its interpretation as a true risk score. After the ERI was calculated for each patient in the cohort, the performance of the index as a predictive tool was rigorously evaluated using standard metrics, including receiver operating characteristic (ROC) curve analysis. This analysis allowed for the determination of an optimal ERI cutoff value (identified as 0.80), which transforms the continuous risk score into the model’s final binary prediction of “endoleak positive” or “endoleak negative,” thereby providing a clear, actionable output for preoperative planning (Figure 1E,F).

### 2.8. Geometric Validation

These metrics primarily validate the initial geometric reconstruction phase, ensuring the digital twin’s physical representation is faithful to the patient’s anatomy. We measured the overlap between the segmented volume from the CTA and the final generated mesh volume. A high dice similarity coefficient > 0.95 confirmed that the model accurately captured the volume of the patient’s abdominal aorta. Also, we measured the maximum difference between the surfaces of the segmented aorta and the generated mesh. A low Hausdorff distance < 0.5 mm indicated that the vessel surfaces were geometrically close, validating the fidelity of the wall boundaries used for the simulation.

### 2.9. Propensity Score Matching and Comparative Analysis

To benchmark the ERI-based digital twin approach against traditional EVAR planning, a propensity score matching (PSM) analysis was performed. The propensity score, representing the probability of a patient belonging to the digital twin cohort based on their baseline characteristics, was estimated using a logistic regression model. The model incorporated key baseline demographic, comorbidity, and anatomical variables known to influence EVAR outcomes and type IA endoleak risk. These included: age, gender, body mass index (BMI), hypertension, diabetes, coronary artery disease, chronic obstructive pulmonary disease (COPD), chronic kidney disease (CKD), chronic renal failure on hemodialysis, cerebrovascular disease, hyperlipidemia, hostile neck anatomy, neck length, neck angle, and neck diameter. Matching was then performed using a 1:3 nearest neighbor matching without replacement algorithm. To ensure the selection of closely matched control subjects and improve covariate balance, a caliper width of 0.2 standard deviations of the logit of the propensity score was applied. The balance of covariates between the matched groups was assessed by calculating standardized mean differences (SMDs) for all included variables, with an SMD value less than 0.1 generally considered indicative of good balance. This PSM approach aimed to minimize selection bias between the groups, allowing for a robust, comparative assessment of clinical outcomes between the digital twin-guided approach and the conventional planning. Our study cohort (*n* = 15) was compared against a control group of patients (*n* = 45, matched 1:3 ratio) who underwent EVAR at our institution during the same period using traditional anatomical measurements for planning (Supplementary Materials).

### 2.10. Predictive Accuracy and Statistical Analysis

To validate the ERI, patients were retrospectively categorized into two groups based on their presence of endoleak after EVAR: 5 patients with a confirmed postoperative type IA endoleak and 10 patients without a type IA endoleak. The mean ERI values for these two groups were compared using a two-tailed *t*-test to determine if there was a statistically significant difference. The predictive accuracy of the ERI was assessed using receiver operating characteristic (ROC) curve analysis. Key performance metrics were calculated at a predefined ERI cut-off value of 0.80. These metrics included sensitivity, specificity, positive predictive value (PPV), negative predictive value (NPV), and overall accuracy. The area under the curve (AUC) was also calculated to provide a comprehensive measure of the model’s discriminatory power in distinguishing between endoleak-positive and endoleak-negative patients.

## 3. Result

### 3.1. Patient Characteristics

The study included a well-defined cohort of 15 patients in the AI model group and 45 patients in the PSM conventional group. The baseline demographic and anatomical characteristics between these two matched cohorts were largely balanced. The mean patient age in the AI group was 78.6 ± 8.7 years, which was statistically identical to the matched conventional group 78.6 ± 9.7 years, *p* = 0.994). Similarly, there were no significant differences in gender distribution (73.3% vs. 75.6% male, *p* = 0.863) or body mass index (22.7 ± 4.7 kg/m^2^ vs. 23.3± 3.6 kg/m^2^, *p* = 0.615). Matching was successful in controlling for hostile neck anatomy, which was present in an equal percentage of patients in both the AI and matched conventional groups (53.3%, *p* = 1.000). However, two significant baseline differences persisted despite the matching process. Patients in the AI group had a significantly higher prevalence of smoking history (86.7%) compared to the matched conventional group (37.8%, *p* = 0.001). Furthermore, the AI group exhibited a substantially higher rate of existing endoleaks (33.3%) than the conventional group (4.4%, *p* = 0.003). Other comorbidities, including hypertension (*p* = 0.103) and coronary artery disease (*p* = 0.178), showed no statistical difference. A detailed breakdown of patient demographics and aneurysm characteristics for both groups is presented in Table 1.

### 3.2. Virtual Deployment Accuracy and Visual Validation

**Figure 3:**
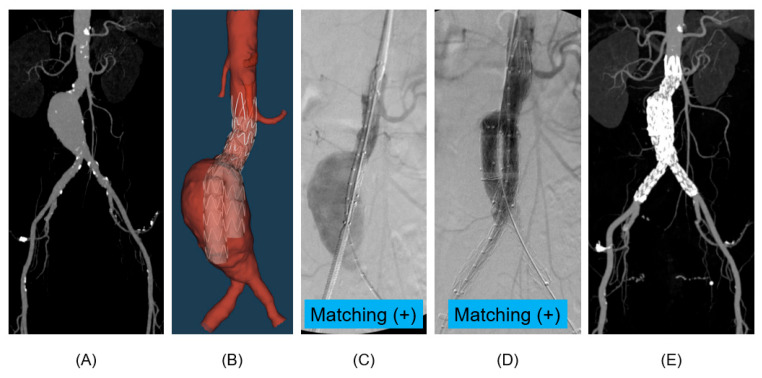
Example of a matching virtual deployment. This figure presents a case where the digital twin model’s prediction accurately matched virtual deployment. (**A**) A preoperative maximal intensity projection image, (**B**) a virtual deployment image using the digital twin model for EVAR simulations, (**C**) matched fluoroscopic image at the proximal stent-graft deployment, (**D**) matched fluoroscopic image at the full deployment of stent-graft, (**E**) postoperative maximal intensity projection image.

**Figure 4:**
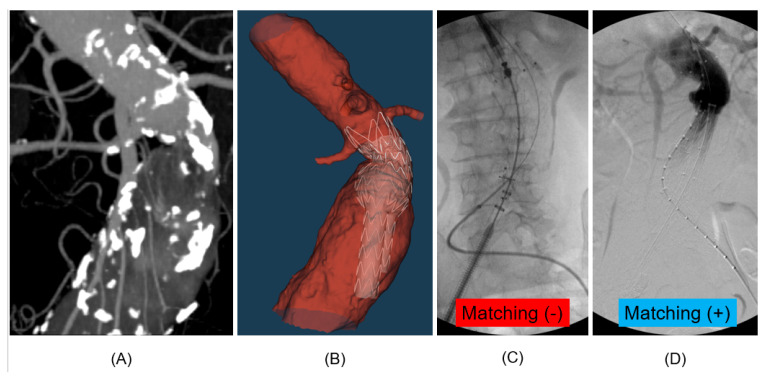
Example of a non-matching virtual deployment. This figure illustrates a case where the virtual deployment simulation did not match at the time of proximal stent-graft deployment. (**A**) A preoperative maximal intensity projection image, (**B**) a virtual deployment image using the digital twin model for EVAR simulations, (**C**) Unmatched fluoroscopic image at the proximal stent-graft deployment, (**D**) matched fluoroscopic image at the full deployment of stent-graft.

Beyond statistical metrics, the study also provided visual confirmation of the digital twin model’s accuracy. The virtual deployment simulations were directly compared with the fluoroscopic images during the procedure and the postoperative CTA imaging to assess the model’s ability to replicate the real deployment of a stent-graft accurately. In a patient without endoleak, the virtual model accurately reproduced the locations and characteristics of the intraoperative fluoroscopic images and postoperative CTA images, as depicted in Figure 3. This precise matching between the virtual simulation and clinical reality provides compelling visual evidence of the model’s validity and reliability. Figure 4 demonstrates that the virtual model fails to match the fluoroscopic images during the procedure. However, the image was fully matched after the full deployment of the stent-graft.

### 3.3. Endoleak Risk Index: A Predictive Metric

A key objective of this study was to validate the utility of the ERI as a preoperative predictor for type IA endoleaks. The digital twin model, created for each patient, calculated a unique ERI based on patient-specific aortic geometry, biomechanical properties of the aortic wall, and the material and geometric properties of the planned stent-graft. The primary finding of our analysis was the identification of a statistically significant difference in the ERI between the two patient groups (Table 2). The mean ERI was found to be significantly higher in the group that developed a postoperative endoleak compared to the group that did not (*p* = 0.011). This finding, with a *p*-value well below the standard threshold of 0.05, provides strong statistical evidence that the ERI is not only different between the two groups but is also a robust indicator of endoleak risk. The mean ERI for the positive endoleak group was calculated to be 0.85 ± 0.10, while the mean ERI for the negative endoleak group was found to be 0.39 ± 0.11. This significant separation in ERI values between the two groups demonstrates the model’s ability to quantitatively differentiate between high-risk and low-risk patients before the procedure. The clinical implication of this finding is profound. By providing a numerical risk score, the digital twin model allows for a more objective assessment of a patient’s likelihood of developing a type IA endoleak. This could potentially inform surgical planning and patient selection, allowing for the pre-emptive adoption of mitigating strategies, such as the use of an oversized stent-graft or adjunctive endovascular techniques, to prevent endoleaks from occurring.

### 3.4. Predictive Accuracy of the Digital Twin Model

The predictive performance metrics for the AI model and the conventional approach are summarized in Table 3. Overall, the AI model demonstrated superior accuracy compared to both the pre-matched and post-matched conventional groups. The AI model achieved a total accuracy of 80% (95% CI: 51.9–95.7%), significantly higher than the matched conventional group, which achieved only 51.1% (95% CI: 35.7–66.3%). This superior discriminatory power was reflected in the area under the curve (AUC), where the AI model scored 0.85 (95% CI: 0.58–0.99), indicating good diagnostic capability, whereas the matched conventional model scored 0.54 (95% CI: 0.39–0.69), suggesting performance near that of random chance. A key strength of the AI model was its perfect ability to identify positive outcomes, achieving a sensitivity of 100% (95% CI: 47.8–100%). In contrast, the matched conventional model had a very low sensitivity of 8.3% (95% CI: 1.0–27.0%). This difference resulted in a dramatic difference. The AI model’s high sensitivity, coupled with a specificity of 70.0% (95% CI: 34.8–93.3%), resulted in a perfect negative predictive value of 100% (95% CI: 59.0–100%). Conversely, the conventional model achieved perfect specificity of 100% (95% CI: 83.9–100%). Due to its high specificity and low event rate, the conventional model achieved a perfect positive predictive value of 100% (95% CI: 15.8–100%).

## 4. Discussion

The findings of this study validate the clinical utility of a digital twin and AI model as a transformative tool for the preoperative prediction of type IA endoleak following EVAR. Traditionally, conventional planning has relied heavily on static anatomical measurements derived from CTA and a surgeon’s subjective judgment. The most compelling finding is the model’s remarkable overall predictive accuracy of 80% (95% CI: 51.9–95.7%) in our cohort of 15 patients, which stands in sharp contrast to the mere 51.1% accuracy and AUC of 0.54 achieved by the matched traditional planning group. This result is a substantial leap forward from traditional EVAR planning methods. By integrating patient-specific biomechanics and simulating the dynamic interaction between the stent-graft and the aortic wall, this model overcomes the limitations of traditional planning and provides a level of predictive insight previously unattainable [8].

The high predictive accuracy in both endoleak-negative (70.0%) and endoleak-positive (100%) groups holds significant clinical implications. For patients predicted to be endoleak-negative, this technology can provide crucial preoperative reassurance, potentially leading to more streamlined procedures and reducing the need for extensive postoperative surveillance in low-risk cases. Conversely, for the endoleak-positive group, the model correctly identified in all cases. This high positive predictive value means that clinicians can identify high-risk patients before the procedure even begins. This early identification enables proactive surgical planning, such as considering an alternative stent-graft design, adjusting the sizing, or even opting for a different therapeutic approach altogether [15]. By shifting the detection of risk from a post-procedural complication to a preoperative prediction, this technology has the potential to fundamentally alter the EVAR workflow, enhancing patient safety and improving long-term outcomes [16].

A particularly noteworthy finding is the discrepancy between the virtual deployment matching rates at the proximal deployment (41.2%) and full deployment (94.1%) stages. The low matching rate at the initial proximal deployment reflects the significant influence of procedural factors, such as the exact moment and position of the stent-graft release, which can vary between surgeons and even within the same procedure. The aorta itself, being a dynamic structure, also exhibits immediate compliance upon device release, contributing to this initial variability [17]. However, the high matching rate at full deployment is the most clinically relevant metric. The final, fully expanded configuration of the stent-graft is what determines the long-term seal and prevents a type IA endoleak. The model’s ability to accurately predict this final state—despite the initial procedural variability—demonstrates its robustness and its capacity to isolate the most critical biomechanical factors that govern seal integrity. This finding strongly supports the model’s use as a pre-operative planning tool, as it accurately forecasts the outcome that truly matters for patient safety.

Currently, AI, including mobile devices, supports assessing the risk and predicting the prognosis in patients with cardiovascular diseases [18]. It ushers in an era of personalized medicine for EVAR. The study justified its findings by achieving 100% Sensitivity and 100% NPV, establishing it as an excellent safety tool for ruling out type IA endoleak risk. Based on this analysis, the ERI may be used in the clinical workflow as follows. When the ERI is lower than 0.80, the surgeon gains confidence to proceed with a standard EVAR using a conventional device. The 100% NPV suggests that these patients are highly unlikely to experience a type IA endoleak. When the ERI is higher than 0.80, the model flags the proximal neck as anatomically challenging or unsuitable for a standard seal. This mandates escalation or reevaluation of the plan. Despite this strong performance, the small sample size limits generalizability, necessitating larger future studies. The 62.5% PPV was acknowledged as a trade-off. While this could imply potential over-triage for some patients, the primary aim of this POC study was to achieve maximum sensitivity for a critical complication. Future work will focus on refining the model to optimize PPV while retaining high sensitivity, ensuring an ideal balance for clinical decision-making. Computational studies highlight that patient-specific simulations are effective for predicting proximal sealing and endoleak complications, even in complex aortic anatomies, validating the core methodology of digital twin modeling [19]. This technical foundation is critical, as a broader perspective confirms that digital twin technology is transformative for personalized surgery but must overcome significant challenges related to data quality, validation standards, and integration into clinical workflows for widespread adoption [20]. Current device selection is often based on standard anatomical dimensions, which oversimplify the complex three-dimensional relationship between the stent-graft and the aortic neck [11,12,21]. The digital twin model, by contrast, considers a myriad of patient-specific factors, including the unique tortuosity, and the biomechanical properties of the aortic wall [15,22,23]. By simulating different stent-grafts and analyzing their performance within a patient’s unique anatomy, the model could enable a surgeon to select the optimal device and dimensions for each individual case. This personalized approach to surgical planning could minimize the risk of endoleak, reduce the need for secondary interventions, and ultimately lower the long-term healthcare costs associated with EVAR complications. In a healthcare system increasingly focused on value-based care, a tool that can prevent costly re-interventions and improve patient outcomes holds immense economic value [8,24].

This study’s findings must be interpreted within the context of several limitations inherent to its design, which must be addressed prior to clinical adoption. The retrospective design and the small single-center cohort introduce significant patient selection bias, limiting the generalizability of the reported 100% Sensitivity and potentially overestimating the model’s performance across diverse anatomies and surgical practices. Beyond methodological bias, the transition to real-world application faces practical barriers, chiefly the high computational cost and time demands of the underlying fluid structure interaction simulation. For the workflow integration to be viable for elective surgical planning, this duration must be reduced significantly through cloud-based processing. Furthermore, successful real-world deployment requires not only the streamlining of the image to analysis pipeline but also achieving regulatory clearance and benchmarking the tool’s performance against expert human judgment to ensure a clear net benefit in reducing endoleak rates.

While the results of this study are highly promising, it is essential to acknowledge its limitations. The primary limitation is the small sample size of 15 patients. The small sample size limits the statistical power and generalizability of the findings. Therefore, we aimed at retrospective POC validation of the predictive accuracy of a novel digital twin. A key limitation is the absence of prior interventions, specific medication use, and detailed procedural characteristics in our propensity score matching. In addition, two clinically important variables, smoking history and pre-existing endoleak, remained significantly imbalanced between the groups due to inherent differences between the two groups. This omission prevents full control of these potential confounders, potentially influencing comparisons and generalizability, necessitating richer data in future studies. Although the high accuracy in this cohort is a strong indicator, a larger, multicenter study is required to validate these findings and to assess the model’s generalizability across a broader patient population. A larger study would also provide more data on rare clinical cases, such as the single false negative observed in our cohort, allowing for further refinement of the AI algorithm. Furthermore, this was a retrospective study. A future multicenter, prospective study, where the model’s predictions are used to inform surgical planning in real-time and then correlated with clinical outcomes, would provide even more compelling evidence of its clinical utility. Another limitation is the dependence on high-quality pre-operative CTA scans. Variations in image quality or acquisition protocol could impact the accuracy of the digital twin model, and a standardized protocol for imaging acquisition would be necessary for widespread clinical adoption. The computational resources required to create and run these simulations are also a consideration, although advances in hardware and cloud computing are continuously making this technology more accessible. While a formal inter-observer variability study was not conducted in this retrospective cohort, we implemented strict protocols to minimize intra-observer error and ensure data consistency. Also, the absence of a formal inter-observer variability analysis is a limitation. In addition, the current study did not directly quantify operator experience or volume because this study’s output was designed to inform risk management decisions by the operating surgeon.

## 5. Conclusions

This retrospective POC study successfully validated a novel, physics-informed digital twin framework, coupled with an automated CI algorithm, for predicting the risk of type IA endoleak following EVAR. The derived ERI, which integrates specific biomechanical features like SGM, demonstrated significantly superior predictive performance (high AUC, sensitivity, and specificity) compared to conventional static anatomical measurements, such as the aortic neck angle. Crucially, the comparison against a 1:3 propensity-matched control cohort, planned using conventional methods, confirmed the clinical utility and robustness of this dynamic, simulation-based approach. The digital twin might transform EVAR planning from a subjective assessment based on static images to an objective, personalized, and quantitative methodology by providing a high-fidelity simulation of device deployment and precise quantification of seal failure precursors. While the initial cohort of 15 patients confines this study to a validation of feasibility, the established ERI framework is now ready to be leveraged as a robust, validated feature set for training scalable, generalizable clinical predictive AI models in subsequent multi-center trials. Ultimately, the ERI offers a significant pathway toward minimizing post-EVAR complications and optimizing patient-specific treatment strategies.

## Figures and Tables

**Table 1:** Baseline demographics.

Factors	AI Model	Conventional Before Matching		Conventional After Matching	
		Value	*p* Value	Value	*p* Value
Number	15	221	NA	45	NA
Age, year	78.6 ± 8.7	74.6 ± 8.6	0.083	78.6 ± 9.7	0.994
Gender (male)	11 (73.3)	183 (82.8)	0.353	34 (75.6)	0.863
Body mass index, kg/m^2^	22.7 ± 4.7	23.9 ± 3.9	0.241	23.3 ± 3.6	0.615
Risk factors					
Hypertension	14 (93.3)	162 (73.3)	0.085	33 (73.3)	0.103
Diabetes	1 (6.7)	48 (21.7)	0.164	7 (15.6)	0.380
Coronary artery disease	1 (6.7)	44 (19.9)	0.206	10 (22.2)	0.178
COPD	2 (13.3)	12 (5.4)	0.210	3 (6.7)	0.418
CKD	1 (6.7)	17 (7.7)	0.885	3 (6.7)	1.000
CRF on hemodialysis	1 (6.7)	10 (4.5)	0.703	2 (4.4)	0.732
Cerebrovascular disease	3 (20.0)	46 (20.8)	0.940	11 (24.4)	0.724
Hyperlipidemia	3 (20.0)	28 (12.7)	0.416	6 (13.3)	0.531
Smoking history	13 (86.7)	88 (39.8)	<0.001	17 (37.8)	0.001
Hostile neck anatomy	8 (53.3)	64 (29.0)	0.047	24 (53.3)	1.000
Endoleak (+)	5 (33.3)	12 (5.4)	<0.001	2 (4.4)	0.003
Neck length	39.0 ± 10.1	32.8 ± 16.9	0.282	28.4 ± 16.6	0.077
Neck angle	55.4 ± 43.8	36.6 ± 22.1	0.019	45.3 ± 23.3	0.357
Neck diameter	21.2 ± 2.5	21.2 ± 3.3	0.982	21.8 ± 5.5	0.745

NA = not applicable; COPD = chronic obstructive pulmonary disease; CKD = chronic kidney disease (Cr ≥ 2.0 mg/dL); CRF = chronic renal failure. Data are presented as numbers (%) or mean ± standard deviation. For continuous variables, the independent samples *t*-test was used. And the Mann–Whitney U test was used if the *t*-test was not met. For categorical variables, the Chi-squared test was used. And Fisher’s exact test was used where cell counts are low.

**Table 2:** Accurate matching of the virtual deployment, endoleak risk index, and prediction of the type IA endoleak.

Variables	Matching When Proximal Deployment	Matching at the Full Deployment	Accurate Prediction of Endoleak	Endoleak Risk Index ^1^
Endoleak (−) (*n* = 10)	5 (50.0)	10 (100.0)	7 (70.0)	0.39 ± 0.11
Endoleak (+) (*n* = 5)	2 (40.0)	4 (80.0)	5 (100.0)	0.85 ± 0.10
Total (*n* = 15)	7 (46.7)	14 (93.3)	12 (80.0)	0.54 ± 0.36

Data are presented as numbers (%) or mean ± standard deviation. ^1^ The endoleak risk index was significantly higher in the positive endoleak group compared to the negative endoleak group (*p* = 0.011).

**Table 3:** Predication accuracy.

Statistic	Artificial Intelligence Model	Conventional Before Matching	Conventional After Matching
Sensitivity	100% (47.8–100)	10.9 (4.5–21.2)	8.3 (1.0–27.0)
Specificity	70.0% (34.8–93.3)	96.8 (92.7–98.9)	100 (83.9–100)
Area under the curve	0.85 (0.58–0.99)	0.54 (0.47–0.61)	0.54 (0.39–0.69)
Positive likelihood ratio	3.33 (1.29–8.59)	3.43 (1.13–10.42)	None
Negative likelihood ratio	0	0.92 (0.84–1.01)	0.92 (0.81–1.03)
Positive predictive value	62.5% (39.3–81.1)	58.3 (31.6–80.9)	100 (15.8–100)
Negative predictive value	100% (59.0–100)	72.7 (70.9–74.4)	48.8 (45.8–51.9)
Accuracy	80% (51.9–95.7)	71.9 (65.5–77.8)	51.1 (35.7–66.3)

Data are presented as a value (95% confidence interval).

## Data Availability

The original contributions presented in this study are included in the article/Supplementary Materials. Further inquiries can be directed to the corresponding author.

## Supplementary Materials

The following supporting information can be downloaded at: https://www.mdpi.com/article/10.3390/bioengineering13010001/s1, Table S1: After matching; Table S2: Before matching; Table S3: PSM characteristics.
